# Stacked human aortic endothelial cells induce atherosclerotic fatty streaks and release proinflammatory cytokines and chemokines^☆^

**DOI:** 10.1016/j.mbm.2026.100192

**Published:** 2026-04-27

**Authors:** Ye Zeng, Zhi Ouyang, Yan Qiu, Wenli Jiang, Chen Jin, Jian Zhong, Linlu Jin, Yixue Qin, Yunran Zhao, Xintong Zhou, Xiaoheng Liu, Bingmei M. Fu

**Affiliations:** a Institute of Biomedical Engineering, West China School of Basic Medical Sciences & Forensic Medicine, Sichuan University, Chengdu, 610041, China; b Department of Biomedical Engineering, The City College of the City University of New York, New York, 10031, USA

**Keywords:** Cytokine, Endothelial cell, Fatty streak, Intimal injury

## Abstract

Fatty streaks, one of the earliest signs of atherosclerosis, comprise lipid-laden foam cells, which are traditionally thought to be derived from circulating monocytes that have infiltrated through the leaky endothelium and from vascular smooth muscle cells that have migrated into the intima from the media. Here we demonstrate that fatty streaks can also be induced by stacked human aortic endothelial cells (HAECs). Scanning electron microscopy revealed that these stacked HAECs form a new cellular phenotype presenting a streak/coral-like structure: “coralthelial cells”. These coralthelial cells exhibit considerable lipid accumulation and increased expression of Golgi and coat protein II markers in their nuclei. Moreover, proinflammatory mediators, including interleukin-6, monocyte chemoattractant protein-1, and C-X-C motif chemokine ligand 8, are upregulated in the coralthelial cell, potentially due to the nuclear translocation of the Golgi apparatus and an elevated expression of the ribosomal protein RPL23 in the nucleolus. To our knowledge, we demonstrate for the first time that fatty streak–like structures found in atherosclerotic plaques can also be generated from the stacked HAECs.

## Graphical abstract

**Figure:**
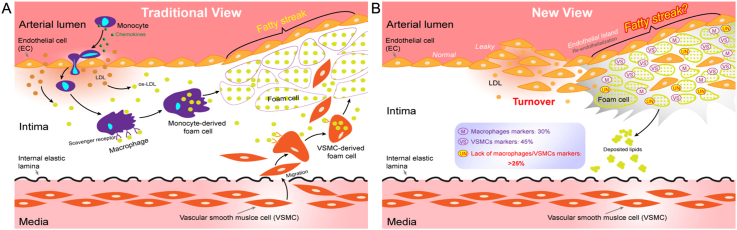

## 1 Introduction

**Fig. 1:**
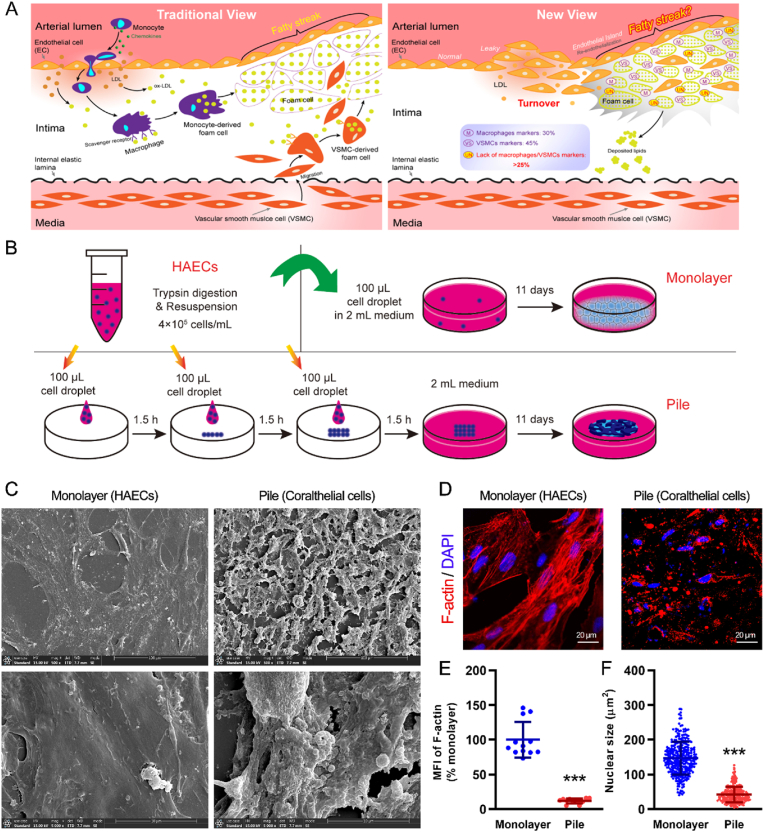
**Origin of fatty streaks and their formation from coralthelial cells transformed from stacked human aortic endothelial cells (HAECs).** (A) Schematic of the traditional and current views regarding fatty streak formation. Traditional view (left): Circulating monocytes are recruited by inflammatory chemokines to sites of endothelial damage, where they enter the intima along with fluid, lipids, and other circulating substances. The accumulated low-density lipoprotein (LDL) in the intima can undergo oxidation and be avidly taken up by monocyte-derived macrophages and vascular smooth muscle cells (VSMCs) migrating into the intima from the media to form macrophage-derived and VSMCs-derived foam cells, respectively. The fatty streak–like structure comprises these lipid-containing foam cells in the arterial wall beneath the endothelium. Current view (right): The initial lipid deposition occurs in the deeper intimal layers proximal to the internal elastic lamina, not in the leaky endothelium or region dominated by macrophage-derived foam cells. The intima covered by the regenerated endothelium is considerably thicker and more likely to accumulate lipids. Surprisingly, approximately 25% or more of the foam cells in regions of thickened intima neither express macrophagic nor VSMC markers. Therefore, these foam cells are thought to be derived from ECs that originate from the rapid turnover after vessel wall damage and get stacked by the local flow in atherosclerosis-prone areas to form an endothelial island. Stacked ECs in the intima can transform into lipid-laden cells, depositing lipids in the deep intimal layers along with the lipid-laden foam cells derived from macrophages and VSMCs. To test this hypothesis, we stacked HAECs in a culture dish to simulate the *in vivo* condition. (B) Schematic showing the generation of monolayer and coralthelial cells (pile) from HAECs. After culturing for 11 days, the surface ultrastructure of the HAEC monolayer and HAEC stack was assessed by SEM (C), and the distribution of F-actin cytoskeleton and nuclei was determined by confocal microscopy (D). In (C), the bottom panels are close-up views of the top panels. (E) MFI (Mean Fluorescence Intensity) of F-actin (averaged over 12 fields of 160 × 160 μm^2^ in each case) and (F) nuclear size (n = 300 for each case) were measured from the confocal images. Data are presented as the mean ± SD; ∗∗∗P < 0.001.

Atherosclerosis is a pathological condition that develops when fatty streaks comprising fats, cholesterol, and cells get deposited inside arterial walls.^1^^,^^2^ Atherosclerosis-associated cardiovascular diseases represent the leading cause of death worldwide. One of the initiating events in the pathogenesis of atherosclerosis is the dysfunction of endothelial cells (ECs), covering the luminal surface of arterial walls.^3^ Endothelial dysfunction or endothelial injury increases endothelial permeability and enhances production of proinflammatory cytokines and adhesion molecules, which cause circulating monocytes to adhere to the endothelial wall and undergo transmigration. The transmigrated monocytes differentiate as macrophages, which, after engulfing low-density lipoproteins (LDLs) accumulated in the intima through the leaky endothelium, get converted to lipid-laden foam cells.^4^^,^^5^ Foam cells secrete various cytokines, triggering an inflammatory reaction. In response, vascular smooth muscle cells (VSMCs) in the arterial media migrate into the intima, in which they proliferate and produce collagen and proteoglycan-rich extracellular matrix.^6^, ^7^, ^8^ Similar to macrophages, VSMCs can turn into lipid-laden foam cells through LDL uptake.^9^ These foam cells are a major component of fatty streaks, and their accumulation substantially contributes to intimal thickening.^9^, ^10^, ^11^ This conventional view of fatty streak formation and cell types in an atherosclerotic plaque is depicted in Fig. 1A.

Initial lipid deposition occurs immediately above the internal elastic lamina, rather than near the leaky endothelium or in the region dominated by macrophage foam cells.^12^, ^13^, ^14^ EC-like cells have been observed in the deep intimal regions.^9^ In a study on normocholesterolemic and hypercholesterolemic rabbits whose aortas were initially de-endothelialized, lipid accumulation was significantly greater in the re-endothelialized intima than in adjacent regions lacking endothelium. Intimal thickness in the re-endothelialized areas, but not in areas lacking endothelium, was enhanced in hypercholesterolemic rabbits compared with normocholesterolemic ones.^11^ The thickened intima covered by the regenerated endothelium was termed “endothelial island”, where increased accumulation of lipids and lipid-laden foam cells was observed (Fig. 1A).

Traditionally, foam cells in the atherosclerotic intima originated from either macrophages or VSMCs,^15^ however, only approximately 30% and 45% of foam cells in atherosclerotic plaques expressed canonical macrophage and VSMCs markers, respectively,^16^ suggesting that foam cells have other cellular origins, besides macrophages and VSMCs. More recently, Allahverdian et al.^17^ showed that 50% of foam cells within advanced human coronary artery lesions expressed the VSMC marker α-smooth muscle actin, but most of these cells expressed the macrophage marker CD68, accounting for 40% of all CD68^+^ cells in the lesion. Using flow cytometry, Wang et al.^18^ revealed that in the atherosclerotic plaques of ApoE^−/−^ mice fed a normal diet for 27 weeks, 37% of foam cells, identified as cells labeled with boron-dipyrromethene (BODIPY), did not express the monocyte marker CD45; this proportion increased to 70% in mice fed a high-fat diet for 6 weeks.

The above findings suggest that approximately 25% of the foam cells in the atherosclerotic intima have cellular origins different from monocytes and VSMCs. Hypothetically, these foam cells are derived from ECs. Single-cell transcriptome sequencing of human atherosclerotic plaques revealed the presence of multiple cell clusters, including ECs, VSMCs, and macrophages,^19^^,^^20^ suggesting that ECs contribute to foam cell formation in fatty streaks. However, the origin of these ECs remains unclear. Although fatty streaks are randomly distributed throughout the arterial tree, early lesions are frequently found at branches, bifurcations, and curves in the circulation where changes in blood flow occur, such as decreased flow, back currents, or eddy currents.^10^^,^^21^^,^^22^ These observations suggest that ECs associated with foam cells originate from the rapid turnover after endothelial injury, getting stacked in atherosclerosis-prone areas to form an endothelial island. The stacked ECs in the intima can transform into lipid-laden cells, depositing lipids in the deeper intimal layers along with the other lipid-laden foam cells derived from macrophages and VSMCs (Fig. 1A).

This study thus tested the aforementioned hypothesis by stacking human aortic ECs (HAECs) in a culture dish to mimic the *in vivo* stacking arrangement observed at sites of disturbed flow. After 11 days, the morphology of the stacked cells was observed by scanning electron microscopy (SEM). Surprisingly, instead of the cobblestone appearance typical of HAEC monolayers, these cells showed a coral-like structure, prompting us to coin the term “coralthelial cells.” Subsequently, we examined the organelles of coralthelial cells using transmission electron microscopy (TEM) and assessed lipid accumulation, endothelial markers, and the expressions of the F-actin cytoskeleton and coat protein complex II (COPII) using immunostaining. The associated chemokines/cytokines and signaling mechanisms of the coralthelial cells were investigated by RNA sequencing. The secretion of the key identified chemokines/cytokines in the cell culture supernatant was evaluated by enzyme-linked immunosorbent assay. Understanding whether such a mechanobiological link exists between endothelial cell stacking, organelle redistribution, and inflammatory activation may offer new insights into the pathogenesis of atherosclerosis and other vascular diseases.

## 2 Results

### 2.1 Surface ultrastructure and actin cytoskeleton distribution of HAECs and coralthelial cells

By stacking HAECs before culturing them, we obtained cells with a distinct phenotype termed coralthelial cells (Fig. 1C). The stacked HAECs were spatially confined to an elongated ridge-like region without covering the entire culture dish, thus completely avoiding an over-confluent state. To further rule out potential confounding effects of nutrient depletion during the 11-day continuous culture, we evaluated cellular senescence and apoptosis. As shown in Fig. S1, the stacked cells formed a localized morphology, leaving peripheral areas empty; neither the monolayer nor the stacked HAECs exhibited obvious senescence (SA-β-gal) or apoptosis (7-AAD). This confirms that the observed phenotypic transformations were driven by the spatial 3D stacking cues rather than compromised cell viability. Fig. 1C shows the surface ultrastructure of the HAEC monolayer and the coralthelial cell stack revealed by SEM (scanning electron microscopy). After 11 days of culture, the surface of the HAEC monolayer appeared smooth, with a few microvilli, small pits, and slender cytoplasmic processes randomly distributed over the surface. In contrast, the stacked HAECs displayed a three-dimensional growth, and stigmata (circular loops) and coral-like structures. These piled cells possess an elongated ridge-like morphology containing a large population of small, spherical, rough blebs on their surface (hence named “coralthelial cells”). The morphology of these cells is very different from the typical morphology of ECs grown in a monolayer. Cells with a similar coral-like morphology have been presented in hepatic sinusoidal walls, where they are regarded as “fat-storing cells”,^23^ and also in aortic regions with focal swelling in rabbits maintained on a cholesterol-rich diet.^24^

**Fig. 2:**
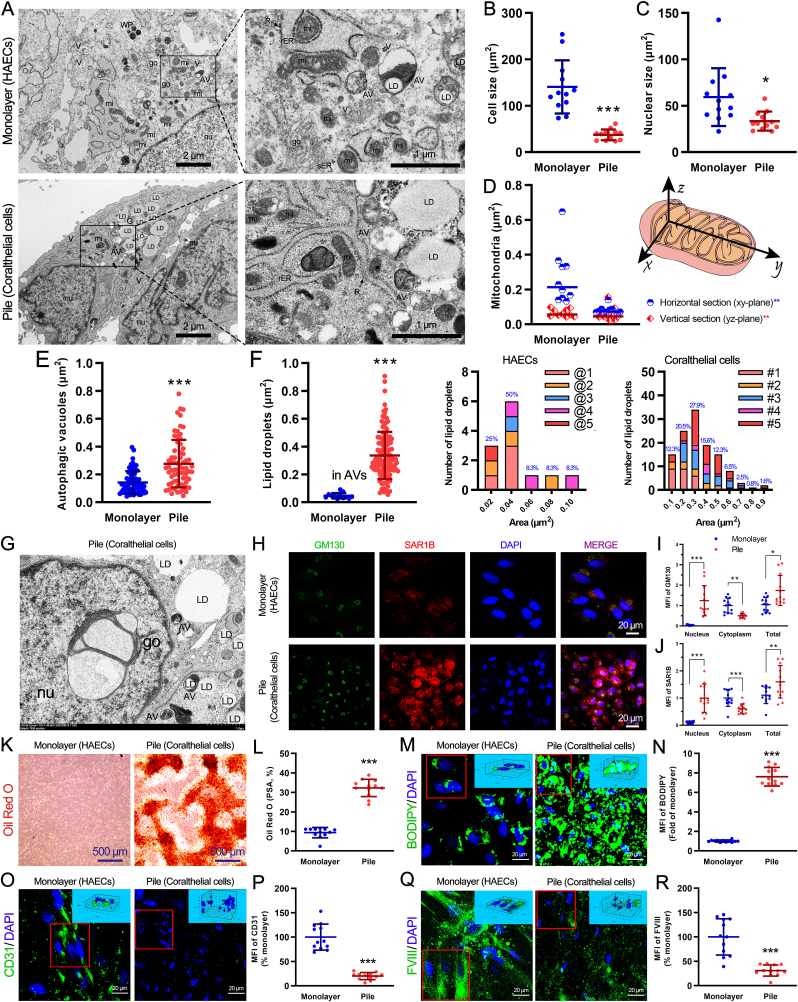
**Ultrastructure of coralthelial cells, nuclear import of Golgi, lipid deposition, and endothelial marker status.** (A) After culturing monolayer HAECs and coralthelial cells for 11 days, their ultrastructure and cytoplasmic organelles were assessed by TEM. Size quantification of cells (B), nuclei (C), mitochondria (D), autophagic vacuoles (E), and lipid droplets (F). (G) Ultrastructure and location of the Golgi apparatus in coralthelial cells. (H) Monolayer HAECs and coralthelial cells were co-stained with anti-GOLGA2/GM130 and -SAR1B antibodies. The MFIs of GM130 (I) and SAR1B (J) in the nucleus, cytoplasm, and overall are presented as fold-changes relative to their MFIs in the cytoplasm per cell in the HAEC monolayer. Lipid droplet deposition was assessed by staining with Oil Red O (K) and BODIPY lipid probes (M). (L) and (N) show the corresponding positive staining area (PSA) in terms of percentage (%) or MFI. Cells were also stained for the endothelial markers CD31 (O) and FVIII (Q). (P) and (R) show the corresponding MFI values. The 3D views in the right top corner in (M, O, and Q) correspond to the red boxed regions in these panels. WP, Weibel–Palade body; V, pinocytic vesicles; go, Golgi apparatus; mi, mitochondria; AV, autophagic vacuoles; LD, lipid droplet; G, glycogen; nu, nucleus; R, ribosomes; rER, rough endoplasmic reticulum; sER, smooth endoplasmic reticulum; HAECs, human aortic endothelial cells; TEM, transmission electron microscopy; MFI, mean fluorescence intensity; BODIPY, boron-dipyrromethene; FVIII, factor VIII; SD, standard deviation. Data are presented as mean ± SD; ∗P < 0.05, ∗∗P < 0.01, ∗∗∗P < 0.001.

**Fig. 3:**
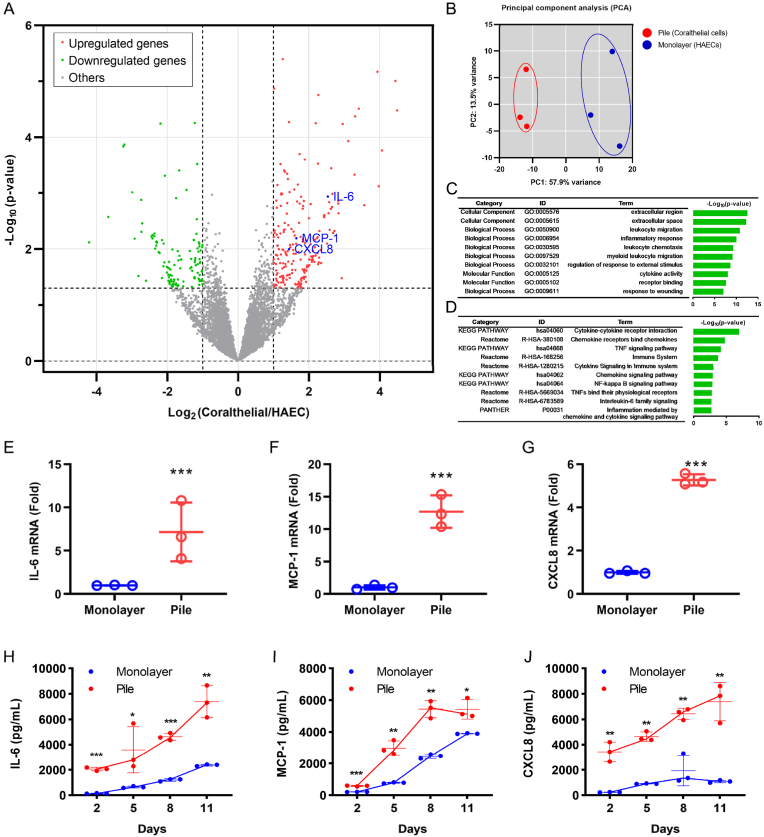
**RNA sequencing analysis of coralthelial cells and chemokines/cytokines detection.** (A) Volcano plot of DEGs. The red and green spots indicate up- and down-regulated genes, respectively. The chemokines/cytokines IL-6, MCP-1, and CXCL8, which plays a role in human cardiovascular events, are marked in blue. (B) PCA based on the transcriptome profile indicates that the first principal component (PC1) can separate coralthelial cells (stack) from HAECs (monolayer). (C) GO analysis of DEGs indicates that the significantly enriched GO terms in coralthelial cells are related to chemokine/cytokine signaling in inflammation. (D) Pathway analysis of DEGs corroborates that chemokine/cytokine signaling pathways in inflammation are significantly altered in coralthelial cells. (E–G) qRT-PCR confirms increased mRNA expression of IL-6 (E), MCP-1 (F), and CXCL8 (G) in coralthelial cells. (H–J) ELISA detection of IL-6 (H), MCP-1 (I), and CXCL8 (J) in the culture medium of coralthelial cells and that of HAECs on days 2, 5, 8, and 11. ∗P < 0.05; ∗∗P < 0.01; ∗∗∗P < 0.001 vs. HAECs (monolayer).

Fig. 1D presents confocal images depicting the distribution of the F-actin cytoskeleton and nuclei in monolayer HAECs and stacked coralthelial cells. In the monolayer, actin stress fibers were organized in long bundles oriented along the cell extensions or lamellipodia. In contrast, the actin fibers in coralthelial cells were sparse, disorganized, amorphous, and slightly granular in some cytoplasm areas. In addition, the mean fluorescence intensity (MFI) of F-actin in coralthelial cells was only 11.75% ± 3.60% of that in the monolayer (Fig. 1E). Therefore, the disorganization of the actin network and actin losses form coralthelial cells. Furthermore, 3D views and orthogonal views derived from confocal Z-stack imaging revealed a clear vertical overlap of nuclei in the pile group, confirming the formation of a three-dimensional stacked architecture, in contrast to the strictly monolayer distribution observed in control HAECs (Fig. 2M-Q and Fig. S1 and Fig. 3). The nuclear size of coralthelial cells was significantly smaller than that of monolayer HAECs (42.16 ± 22.63 vs. 147.10 ± 47.78 μm^2^; Fig. 1F). Compared with monolayer HAECs, coralthelial cells showed reduced amounts of surface glycosaminoglycans but increased amounts of proteoglycans, glycogen, collagens, and calcium, all of which can form an extracellular matrix scaffold, facilitating the assembly of coralthelial cells into fatty streak–like structures (Fig. S1 and Fig. 3).

### 2.2 Coralthelial cells have more and larger lipid droplets and autophagic vacuoles but smaller cell bodies, nuclei and mitochondria

Fig. 2A presents TEM views of cell organelles along with their size quantification in the HAEC monolayer and coralthelial cells. Monolayer HAECs contained Weibel–Palade bodies, ribosomes, rough and smooth endoplasmic reticulum (ER), Golgi apparatus, and mitochondria in an amorphous cytoplasmic matrix surrounding the nucleus (Fig. 2A). Mitochondria appeared adjacent to the rough ER, whereas Golgi complexes appeared adjacent to the smooth ER. Extensive pinocytic vesicles were observed near cell junctions and autophagic vacuoles. The morphology of coralthelial cells and their organelles significantly differed from those of monolayer HAECs (Fig. 2A). The cell body and nucleus were smaller (Fig. 2B and C), and the mitochondria were also smaller, especially in horizontal sections (Fig. 2D). Coralthelial cells lack Golgi apparatus and smooth ER in their cytoplasm, and the number of pinocytic vesicles was dramatically reduced near the junctions (Fig. 2A).

The autophagic vacuoles were filled with moderately electrodense flocculent matrix and lipid droplets (Fig. 2A). It seems that coralthelial cells contained larger autophagic vacuoles (Fig. 2A–E). Surprisingly, although monolayer HAECs exhibited a few lipid droplets, which only appeared in autophagic vacuoles, however, coralthelial cells showed much more and larger lipid droplets (Fig. 2A–F). On average, the amount of lipids per cell in coralthelial cells was approximately tenfold higher than that in monolayer HAECs.

### 2.3 Translocation of Golgi complexes from cytoplasm to nucleus in coralthelial cells

Lipids are secreted by the smooth ER and Golgi apparatus.^25^^,^^26^ Therefore, coralthelial cells, with their higher amount of lipids, are expected to have more smooth ERs and more developed Golgi apparatuses. However, coralthelial cells showed reduced amounts of Golgi and smooth ERs in their cytoplasm compared to monolayer HAECs. To investigate whether Golgi complexes are translocated from the cytoplasm to the nucleus, we carefully checked the localization of Golgi from the TEM images and observed that Golgi complexes do exist in the nuclei of coralthelial cells (Fig. 2G). This observation was validated through immunofluorescence staining of the Golgi marker GOGLA2/GM130 (Fig. 2H). The amount of GOGLA2/GM130 was lower in the cytoplasm but greatly increased in the nucleus of coralthelial cells, yielding a higher amount of overall GOGLA2/GM130 per cell in coralthelial cells (Fig. 2I).

In eukaryotes, transport of newly synthesized lipids and proteins^26^ from the ER to Golgi apparatus is typically mediated by COPII. Thus, we assessed COPII expression through immunofluorescence staining of its marker SAR1B. Consistent with the results for GOGLA2/GM130, overall SAR1B expression was higher in coralthelial cells than in monolayer HAECs (Fig. 2H–J). GM130 and SAR1B were almost absent in the nucleus of monolayer HAECs, whereas their expression was increased in the nucleus and reduced in the cytoplasm of coralthelial cells (Fig. 2H–J), suggesting the nuclear translocation of Golgi protein GOGLA2/GM130 by COPII. Additionally, SAR1B and GOGLA2/GM130 were found to colocalize in the nucleus of coralthelial cells (Fig. 2H).

### 2.4 Lipid accumulation and endothelial marker status in coralthelial cells

Lipid accumulation in coralthelial cells was further verified through Oil Red O and BODIPY staining (Fig. 2K–N). Coralthelial cells displayed a much higher Oil Red O–positive area than monolayer HAECs (32.28 ± 4.43 vs. 9.35 ± 2.64; Fig. 2L). BODIPY-labeled fatty acids appeared as agglomerated, irregular, spherical particles in coralthelial cells (right figure in Fig. 2M), and their mean fluorescence intensity was 7.61 ± 0.96-fold higher than that in monolayer HAECs (Fig. 2N).

Next, we assessed the expression of two common endothelial markers, namely CD31 (PECAM-1) and coagulation factor VIII (FVIII, F8), in monolayer HAECs and coralthelial cells (Fig. 2O-R). Notably, compared with monolayer HAECs, CD31 and FVIII were significantly decreased in coralthelial cells to 19.95% ± 7.28% (Figs. 2P) and 30.97% ± 11.57%, respectively (Fig. 2R). Despite their HAEC origins, coralthelial cells are rich in lipids and poor in EC markers, suggesting that they have been transformed from the HAEC phenotype to a different phenotype with similar characteristics as the foam cell.^27^

### 2.5 Upregulation of inflammation-associated genes and secretion of proinflammatory cytokines in coralthelial cells

To elucidate the molecular mechanisms governing the generation of coralthelial cells from HAECs, we performed RNA sequencing, identifying 339 differentially expressed genes (DEGs) between coralthelial cells and HAECs (Fig. 3A). Of the 339 DEGs, 221 were upregulated and 118 downregulated in coralthelial cells. The upregulated genes included those encoding interleukin-6 (IL-6), monocyte chemoattractant protein-1 (MCP-1), and C-X-C motif chemokine ligand 8 (CXCL8), which are proinflammatory cytokines/chemokines that play key roles in human cardiovascular events^28^^,^^29^ (Fig. 3A). Principal component analysis based on the DEGs indicated that the first principal component (PC1: 57.9%) separated coralthelial cells from HAECs (Fig. 3B).

Gene Ontology (GO) analysis of the DEGs revealed that the top enriched cellular components were “extracellular region” and “extracellular space”; the top enriched biological processes were “leukocyte migration,” “inflammatory response,” “leukocyte chemotaxis,” “myeloid leukocyte migration,” “regulation of response to external stimulus,” and “response to wounding”; and the top enriched molecular functions were “cytokine activity” and “receptor binding” (Fig. 3C). Therefore, coralthelial cells exhibit altered chemotaxis and inflammatory responses. Consistent with the results of GO analysis, pathway analysis of the DEGs indicated that the top enriched pathways encompassed four KEGG terms, namely “cytokine–cytokine receptor interaction,” “TNF signaling,” “chemokine signaling,” and “NF-κB signaling”; five Reactome terms, namely “chemokine receptors bind chemokines,” “immune system,” “cytokine signaling in immune system,” “TNFs bind their physiological receptors,” and “IL-6 family signaling”; and one PANHER term, namely “inflammation mediated by chemokine/cytokine signaling pathway” (Fig. 3D). These enriched pathways suggest that coralthelial cells play a critical role in inflammatory chemokine/cytokine activity.

Using quantitative real-time PCR (qRT-PCR), the mRNA levels of the key proinflammatory cytokine IL-6 and chemokines MCP-1 and CXCL8 were higher in coralthelial cells than in monolayer HAECs by 7.18 ± 3.40-fold (Figs. 3E), 12.71 ± 2.49-fold (Figs. 3F), and 5.28 ± 0.26-fold (Fig. 3G), respectively. The secretion of these cytokines by coralthelial cells was confirmed using enzyme-linked immunosorbent assay. The concentrations of IL-6, MCP-1, and CXCL8 in the culture supernatants of coralthelial cells markedly increased with culture time and were significantly higher than the corresponding concentrations in the culture supernatants of monolayer HAECs from days 2 to 11 (Fig. 3H–J). For example, at day 11, the comparative concentrations of IL-6, MCP-1, and CXCL8 in the supernatants of coralthelial cells vs. monolayer HAECs were 7378.71 ± 1256.92 vs. 2365.56 ± 69.86 pg/mL, 5426.25 ± 617.89 vs. 3897.84 ± 20.93 pg/mL, and 7385.77 ± 1504.66 vs. 1079.0 ± 92.72 pg/mL, respectively.

### 2.6 Roles of Golgi nuclear translocation and RPL23 in the production of proinflammatory cytokines

**Fig. 4:**
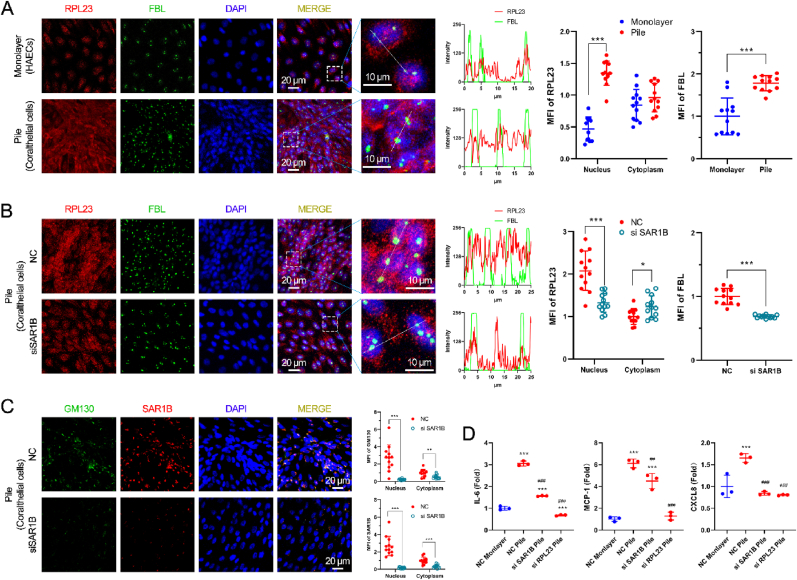
**RPL23 translocates into the nucleolus and facilitates the secretion of proinflammatory cytokines, which is attenuated by SAR1B siRNA.** (A) Co-staining of RPL23 and FBL (the nucleolus marker) in monolayer HAECs and coralthelial cells. (B, C) Following SAR1B knockdown, the nuclear translocation of RPL23 (B) and GM130 (C) were assessed in coralthelial cells. Line scans were performed for RPL23 and FBL. The MFIs of RPL23, FBL, GM130, and SAR1B in the nucleus and cytoplasm are presented as fold-changes relative to their levels in the cytoplasm of monolayer HAECs or NC coralthelial cells (stack). Data are presented as the mean ± SD; ∗P < 0.05, ∗∗P < 0.01, ∗∗∗P < 0.001. (D) The levels of IL-6, MCP-1, and CXCL8 in coralthelial cells were quantified. ∗∗∗P < 0.001 vs. NC monolayer; ##P < 0.01, ###P < 0.01 vs. NC stack.

The ribosomal protein RPL23 is closely associated with production of proinflammatory mediators in ECs and intimal thickening in response to disturbed blood flow.^30^^,^^31^ To investigate the role of RPL23 in shaping a proinflammatory microenvironment, we labeled RPL23 (red) and the nucleolus marker fibrillarin (FBL, green), which is a nucleolar stress response indicator. Coralthelial cells exhibited a disrupted and irregular nucleolar structure compared with monolayer HAECs (Fig. 4A). We observed a remarkable increase in the nucleolar localization of RPL23 in coralthelial cells (red–green overlap), accompanied by increased nucleolar stress, as indicated by the redistribution of fibrillarin (Fig. 4A).

Protein trafficking between the ER and Golgi is essential for cellular processes, including the release of proinflammatory cytokines. To further explore the impact of nuclear translocation of COPII vesicles and Golgi apparatus in coralthelial cells, we attempted to suppress COPII expression through small interfering RNA (siRNA)–mediated knockdown of SAR1B. Strikingly, transfection of siSAR1B did not restore the nucleolar structure but inhibited RPL23 translocation to the nucleolus (Fig. 4B). Thus, siSAR1B may disrupt intranuclear transport in the Golgi apparatus, considering that transport from the ER to the Golgi is typically mediated by COPII. In addition to suppressing the expression of SAR1B, siSAR1B reduced the expression and nuclear translocation of GM130 (Fig. 4C), suggesting that RPL23 is translocated into the nucleus along with GM130. RPL23 participates in 60S subunit assembly^32^ and is located at the exit tunnel of the ribosome, serving as a docking site for the nascent polypeptide-associated complex in protein folding.^33^ Inhibition of Golgi nuclear translocation reduced RPL23 in the nucleus and, consequently, RPL23 in the nucleolus, possibly leading to a decrease in translational activity.

To investigate the requirement of RPL23 for the synthesis of proinflammatory cytokines/chemokines, we examined the secretion of IL-6, MCP-1, and CXCL8 in coralthelial cells transfected with siSAR1B and siRPL23. siSAR1B and siRPL23 significantly suppressed the secretion of these proinflammatory factors (Fig. 4D). Overall, the secretion of chemokines/cytokines by coralthelial cells to create a proinflammatory microenvironment is realized through the nuclear translocation of RPL23 and the Golgi apparatus.

## 3 Discussion

The emergence of fatty streaks is one of the initial signs of atherosclerosis, a condition causatively linked to several clinical morbidities, including coronary heart disease, stroke, and peripheral vascular disease. Fatty streaks in the arterial intima have been traditionally thought to be formed by lipid-laden foam cells derived either from macrophages or VSMCs. In the current study, we presented compelling evidence for the third source of lipid-containing cells, the direct transformation of HAECs into lipid-laden foam cells after stacking them up in culture, without any atherogenic stimuli. Because these transformed cells have a coral-like appearance and originated from endothelial cells, they are thus named as “coralthelial cells”. Similarly shaped cells have been observed in hepatic sinusoidal walls, where they were regarded as “fat-storing cells”,^23^ and in aortic regions with focal swelling in early-stage atherosclerosis.^24^

We observed numerous lipid droplets within coralthelial cells using TEM and the lipid-specific staining techniques of Oil Red O and BODIPY staining. Unlike macrophage- or VSMC-derived foam cells, which are bulkier, coralthelial cells are much smaller than normal HAECs by approximately 25% in cell size and 50% in nucleus size. Although coming from HAECs, coralthelial cells do not express typical endothelial markers CD31 and FVIII, suggesting that they may either evade detection as cells derived from ECs in the atherosclerotic plaques or be misclassified as macrophage- or VSMC-derived foam cells owing to their phenotypic transformation. Coralthelial cells have disorganized cytoskeletons and irregular fatty acids aggregating spherical particles in the cytoplasm. In contrast, there are small and uniformly distributed spherical vesicles in the VSMCs-derived foam cells that are generated by incubating VSMCs with oxidized LDL (ox-LDL).^34^ These distinct cytological features suggest that coralthelial cells represent a new cellular phenotype potentially involved in lipid deposition within atherosclerotic plaques. However, further research is needed to identify specific phenotypic markers for coralthelial cells.

Internalization of natural LDLs by macrophages and VSMCs does not cause intracellular cholesterol accumulation or foam cell formation. The foam cell formation during atherosclerosis has long been associated with the uptake of ox-LDL by macrophages and VSMCs.^35^ Many *in vitro* studies have induced lipid droplet biogenesis and foam cell formation in monocytes or VSMCs through stimulation with high concentrations of ox-LDL (50–100 μg/mL) for 12–72 h.^36^, ^37^, ^38^ Although ox-LDLs are highly cytotoxic to ECs, vascular ECs can take up the active component of ox-LDL, lysophosphatidylcholine (1 μg/mL for 24 h), by phagocytosis.^39^ Hypercholesterolemic serum can transform mouse aortic ECs into foam cells and cause cytoskeletal disorganization.^40^ Instead of using atherogenic agents to induce foam cell formation, we simply achieved this by stacking HAECs in a cell culture dish. Foam cell formation is typically driven by an imbalance in cholesterol influx, esterification, and efflux.^41^ Overexpression of the scavenger receptor lectin-like ox-LDL receptor 1 in ECs induces endothelial dysfunction and exacerbates atherosclerosis in the ApoE^−/−^ mice.^42^ Such metabolic dysfunctions may also transform coralthelial cells, promoting lipid accumulation and subsequent fatty streak formation.

A critical question is whether the *in vitro* induction of lipid-laden coralthelial cells by piling HAECs really occurs *in vivo*. Using SEM, a study reported an increased incidence of lipid-rich fatty streaks in the aortic arch of rabbits fed an atherogenic diet.^43^ The early lesions appear as focal swelling surrounded by morphologically normal endothelium and possibly covered by rounded ECs. Crystal-like structures and small focal swellings have been observed on the luminal surface of aortas in rabbits fed an atherogenic diet, and numerous such focal swellings are hypothesized to constitute early fatty streaks.^43^ Analyses of fresh human atherosclerotic samples have successfully detected lipid and lipid-crystalline droplets within fatty streaks.^44^ These droplets primarily comprise cholesteryl esters and triglycerides, covered with a surface of phospholipids and unesterified (free) cholesterol in the human aorta.^45^ Under *in vivo* conditions, HAECs may undergo rapid turnover following endothelial injuries and get stacked up, possibly by the vascular spasms and local disturbed flow typically at branches, bifurcations, and curves in the vasculature where fatty streaks are initiated.

The recruitment of circulating monocytes and T cells to atherosclerotic sites is driven by proinflammatory cytokines and chemokines. We found that coralthelial cells secreted IL-6, MCP-1, and CXCL8, which are well-documented proinflammatory mediators of atherosclerosis progression. For instance, IL-6 recruits T cells to inflammation sites, whereas IL-8 and MCP-1 are chemotactic agents for leukocytes.^46^^,^^47^ RNA sequencing revealed significant upregulation of inflammatory pathways in coralthelial cells, especially those related to cytokine–cytokine receptor interactions, TNF signaling, and NF-κB signaling. These pathways possibly promote leukocyte migration and exacerbate local inflammation, contributing to the sustained proinflammatory microenvironment observed in atherosclerotic plaques.

A pivotal finding of this study is the nucleolar translocation of the ribosomal protein RPL23, essential for producing proinflammatory cytokines. RPL23 encodes a cytoplasmic ribosomal protein serving as a component of the 60S subunit.^32^ Elevated RPL23 expression is associated with reduced apoptosis in CD34^+^ bone marrow cells, which is related to c-MYC upregulation.^48^ The nucleolus is a critical stress sensor and signaling hub that responds early to cellular damage as nucleolar stress. Impaired incorporation of RPL23 into maturing 60S subunits amplifies nucleolar stress responses under oncogenic stress.^32^ In triple-negative breast cancer cells, RPL23 translocation to the nucleolus triggers c-MYC expression and promotes cellular stemness.^49^ Similarly, we observed concomitant nucleolar stress and increased nucleolar localization of RPL23 in coralthelial cells. SAR1B is a GTP-binding protein involved in the assembly of COPII vesicles and the transport of lipids and proteins from the ER to the Golgi apparatus.^26^ Knockdown of SAR1B and RPL23 significantly reduced cytokine secretion, indicating that RPL23 plays a crucial role in the ER–Golgi–nucleus axis, mediating inflammation in coralthelial cells. RPL23 has also been implicated in intimal thickening in response to disturbed blood flow,^30^^,^^31^ linking mechanical forces, EC stacking, and proinflammatory signaling during atherosclerosis.

Finally, our study underscores the relevance of Golgi–ER dynamics in cellular transformation and inflammation. GOGLA2/GM130 and SAR1B were previously thought to localize exclusively in the cytoplasm of normal ECs.^26^^,^^50^ To our surprise, we observed their unexpected nuclear translocation in coralthelial cells, along with RPL23. These findings suggest that COPII vesicles and the Golgi apparatus, normally restricted to the cytoplasm, translocate to the nucleus in coralthelial cells. Inhibition of the Golgi apparatus through SAR1B knockdown disrupted RPL23 nuclear translocation and proinflammatory factor secretion, underscoring the therapeutic potential of targeting the Golgi–ER axis in atherosclerosis.

**Fig. 5:**
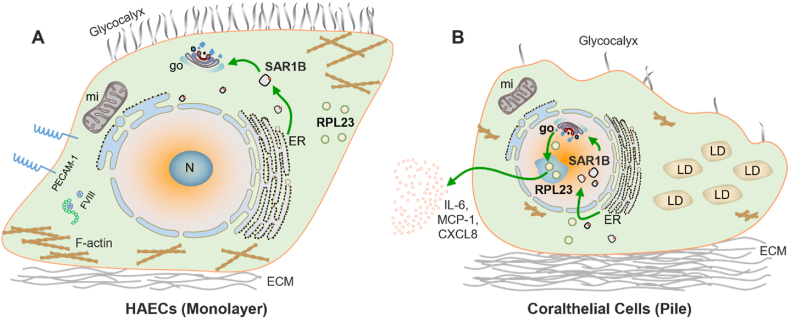
**Major differences between human aortic endothelial cells and coralthelial cells, and the mechanism of cytokine release in coralthelial cells**. (A) HAECs display a typical endothelial morphology with a flattened, elongated structure, organized actin filaments, intact glycocalyx, and minimal lipid accumulation. (B) By contrast, coralthelial cells, derived from stacked HAECs, exhibit a distinct coral-like morphology with surface blebbing, smaller cell bodies and nuclei, disorganized actin filaments, degraded glycocalyx, smaller mitochondria, enhanced nucleolar stress, and prominent lipid droplet accumulation, resembling foam cells found in atherosclerotic plaques. Coralthelial cells also lack typical endothelial markers such as PECAM-1 (CD31) and FVIII, which are expressed in HAECs. Coralthelial cells show a marked increase in collagen production, helping form the extracellular matrix scaffold observed in atherosclerotic fatty streak–like structures. In addition, coralthelial cells exhibit significant upregulation of proinflammatory cytokines and chemokines, including IL-6, MCP-1 and CXCL8, promoting a proinflammatory microenvironment characteristic of atherosclerosis. This cytokine/chemokine release by coralthelial cells is likely driven by the ER–Golgi–nucleus axis and involves the nuclear translocation of RPL23, in conjunction with the Golgi apparatus and COPII vesicles (key component SAR1B). Targeting RPL23 and ER–Golgi dynamics represents a promising therapeutic strategy for modulating cytokine release in vascular diseases, including atherosclerosis. go, Golgi apparatus; ER, endoplasmic reticulum; mi, mitochondria; LD, lipid droplet; N, nucleolus; HAECs, human aortic endothelial cells; PECAM-1, platelet endothelial cell adhesion molecule-1; FVIII, factor VIII; COPII, coat protein complex II; IL-6, interleukin-6; MCP-1, monocyte chemoattractant protein-1; CXCL8, C-X-C motif chemokine ligand 8.

In conclusion, to our knowledge, we demonstrated for the first time that lipid-laden foam cells called coralthelial cells can be directly derived from stacked HAECs without any atherogenic factors. These cells contribute to lipid deposition and create a proinflammatory microenvironment through Golgi nuclear translocation and RPL23 nucleolar expression (Fig. 5), potentially exacerbating plaque formation and disease progression. Our findings present a novel paradigm for understanding atherosclerosis. Future research should investigate the mechanisms driving the transformation of HAECs into coralthelial cells and the potential of targeting Golgi–ER trafficking pathways as a therapeutic strategy for mitigating inflammation and plaque development in atherosclerosis. Furthermore, identifying specific markers for coralthelial cells would facilitate their detection and classification within atherosclerotic plaques, allowing a more accurate understanding of their roles in disease progression. Finally, elucidating the interplay between mechanical forces, EC turnover, and coralthelial cell formation could provide deeper insights into how disturbed flow contributes to the initiation and propagation of fatty streaks and plaques. These investigations can inform the development of novel therapeutic approaches aimed at reducing the burden of cardiovascular disease associated with atherosclerosis.

## 4 Materials and methods

### 4.1 Cell culture

HAECs (#6100; Sciencell, USA) were cultured in a humidified 5% CO_2_ incubator at 37 °C. Upon reaching confluence, the cells were trypsinized with 0.05% trypsin-EDTA (#15400054; Invitrogen, USA) and pelleted by centrifugation at 176×*g* for 5 min in a 3H16RI high-speed centrifuge (HereXi Instrument & Equipment, Hunan, China). The harvested cells were resuspended in fresh endothelial cell medium (#1001; Sciencell, USA) and adjusted to 4 × 10^5^ cells/mL.

TeloHAECs (#CRL-4052; ATCC, USA) were used for siRNA transfection and to investigate the relationship between RPL23 and Golgi nuclear translocation and nucleolar stress. These cells were maintained under the same culture conditions as HAECs, except the endothelial cell medium (#B-SM-00001-500, Biozellen) was used. siRNA-mediated knockdown of SAR1B and RPL23 was performed using Opti-MEM medium and Lipofectamine 3000 reagent, per the manufacturer's instructions. After 48 h of transfection, the cells were subjected to further analysis. Knockdown efficiency was determined by evaluating the mRNA levels of SAR1B and RPL23 using qRT-PCR (Fig. S2). Table S1 details the sequences of the primers used.

### 4.2 Induction of coralthelial cells

For inducing the formation of coralthelial cells, 100 μL of HAEC cell suspension with concentration of 4 × 10^5^ cells/mL was dropped in the center of a 35-mm culture dish (#430165; Corning, USA) and incubated for 1.5 h. After removing nonadherent cells, another 100 μL of cell suspension was dropped at the original position and incubated for 1.5 h. This process was repeated thrice. Finally, after removing nonadherent cells, 2 mL of fresh medium was added to the culture dish, and the adherent cells were cultured for 11 days (HAECs) or 8-9 days (telo-HAECs) at 37 °C in a 5% CO_2_ incubator. Confluent HAECs cultivated into a monolayer served as the control. In total, 100 μL of cell suspension was mixed with 2 mL of fresh medium and cultured for 11 days at 37 °C in a 5% CO_2_ incubator till a monolayer was formed.

### 4.3 Scanning electron microscopy

To evaluate cell surface morphology, monolayer HAECs and coralthelial cells were prepared for SEM. Briefly, the samples were initially washed with phosphate-buffered saline (PBS) (#BL302A; Biosharp, China), fixed overnight in 3% glutaraldehyde (#G810415; Macklin, China) in 0.1 M PBS (pH 7.4), sequentially dehydrated in a graded series of ethanol (30%, 50%, 70%, 80%, 90%, 100%, and 100%) for 15 min each, dried in an EM CPD300 critical point dryer (Leica, Vienna, Austria) for 1.5 h, sputtered with gold using an MC1000 ion sputter coater (Hitachi, Tokyo, Japan), and viewed under a Helios G5 UC field-emission scanning electron microscope (Thermo Fisher Scientific, USA) equipped with an AZtecLive Ultim Max 100 energy-dispersive X-ray spectrometer (Oxford Instruments, UK). SEM was performed at an accelerating voltage of 15 kV.

### 4.4 Cytoskeleton assessment

Polymerized actin filaments were visualized by phalloidin staining. Cells were fixed for 10 min in 4% paraformaldehyde (#P0099; Beyotime, China), washed thrice with PBS, and incubated at room temperature (22-25 °C) with 0.1% Triton X-100 (#ST795; Beyotime, China) for 5 min, followed by the Actin-Tracker Red-555 probe (1:200; #C2203S; Beyotime, China) for 60 min. The cells were washed thrice with PBS again, stained with DAPI (#C1005; Beyotime, China) for 5 min, and finally observed under an LSM 710 confocal laser scanning microscope (Carl Zeiss, Germany) using a Plan-Apochromat 63 × /1.4 oil DIC objective. All quantification was performed using ImageJ (version 1.53q; National Institutes of Health, USA).

### 4.5 Transmission electron microscopy

Cells were initially fixed in 3% glutaraldehyde in 0.1 M PBS (pH 7.4) at 4 °C for 12 h, postfixed in 1% osmium tetroxide in 0.1 M PBS at 4 °C for 2 h, rinsed in distilled water, dehydrated in a graded acetone series (30%, 50%, 70%, 90%, 100%, and 100%) for 10 min each, and embedded in SPI-Pon 812R epoxy resin (#02659R-AB; SPI, USA). The above steps were completed on a glass slide, then the cells were separated from the slide. Ultrathin sections (70 nm thickness) were cut using an EM UC7 ultramicrotome (Leica, Austria), attached to copper grids (AN200, Japan) with Formvar film (Beijing Zhongjingkeyi Technology Co., Ltd., China), stained with 2% uranyl acetate (#02624-AB; SPI) and lead citrate (#02532-BA; SPI) and imaged at 80 kV using a Hitachi HT7800 transmission electron microscope (Tokyo, Japan).

### 4.6 Oil Red O staining and quantification

Lipid accumulation in cells was detected by Oil Red O staining. Cells were washed thrice with PBS, fixed in 4% paraformaldehyde for 10 min, and then washed thrice with deionized water. Oil Red O (#G1262; Solarbio, China) was thoroughly mixed with distilled water in a 3:2 ratio, and the stain solution was filtered through a neutral filter paper (Whatman, USA). After staining with Oil Red O for 30 min, the cells were rinsed with deionized water and observed under an EVOS XL Core inverted phase-contrast microscope (Thermo Fisher Scientific, USA). For quantification, Oil Red O was extracted in 100% isopropanol for 20 min, and absorbance was measured at 510 nm using a SpectraMax 190 full-wavelength micrometer (Molecular Devices, USA).

### 4.7 BODIPY staining

A BODIPY probe was used to visualize cellular lipid droplets. Cells were fixed in 4% paraformaldehyde for 10 min, washed thrice in PBS for 5 min each, stained with 2 μM BODIPY 493/503 (#D3922; Molecular Probes, USA) for 15 min, counterstained with 3 nM DAPI (#D1306; Invitrogen, USA) for 5 min, and observed by confocal laser scanning microscopy.

### 4.8 Immunofluorescence staining of canonical endothelial markers

Cells were fixed in 4% paraformaldehyde for 10 min, washed thrice with PBS, permeabilized with 0.1% Triton X-100 for 5 min, incubated with 1% BSA (#A7284; Sigma Aldrich, USA) in PBS for 20 min, probed with rabbit anti-CD31 and anti-FVIII antibodies (1:200; #CSB-PA017767LA01HU and #CSB-PA007932LA01HU; CUSABIO, China) for 1 h, washed thrice with PBS, incubated at room temperature for 1 h with Alexa 488–conjugated goat antirabbit IgG (1:400; #CA11034s; Invitrogen, USA) followed by DAPI (Invitrogen, USA) for 5 min, washed thrice with PBS, and finally observed by confocal laser scanning microscopy.

### 4.9 Detection of glycocalyx, proteoglycan, collagen, and calcium

Detailed information about these procedures is provided in the Supplementary Methods.

### 4.10 Immunofluorescence staining of Golgi, COPII, RPL23, and fibrillarin

Cells were fixed in 4% paraformaldehyde for 10 min, washed thrice in PBS, permeabilized with 0.1% Triton X-100 for 5 min, incubated in 3% BSA in PBS for 30 min to block nonspecific binding sites, probed with specific primary antibodies at room temperature for 1 h, washed thrice with PBS, incubated at room temperature for 1 h with Alexa Fluor 488–conjugated goat antimouse IgG (1:400; # CA11001s; Invitrogen, USA) or Alexa Fluor 555–conjugated donkey antirabbit IgG (1:400; #A0453; Beyotime, China), stained with DAPI (Invitrogen, USA) for 5 min, washed with PBS, and imaged using a confocal laser scanning microscope. Quantification analyses were performed using ImageJ. The primary antibodies used were as follows: mouse anti-GOLGA2/GM130 (1:200; #AG2041; Beyotime, China), rabbit anti-SAR1B (1:200; #22292-1-AP; Proteintech, USA), rabbit anti-RPL23 (1:200; #A4292; ABclonal, China), and mouse anti-fibrillarin (FBL; 1:200; #66985-1-Ig; Proteintech, USA).

### 4.11 RNA sequencing and identification of chemokines/cytokines

RNA was extracted cells using TRIzol reagent (#15596-0180; Life Technologies, USA) per the manufacturer's instructions, followed by RNA integrity evaluation, library construction for mRNA sequencing, and high-throughput sequencing using an Illumina Genome Analyzer platform (CapitalBio Corp, Beijing, China). The raw reads were initially filtered by removing low-quality reads, filtering contaminants, and trimming adaptor sequences. The unigenes resulting from the assembly of mRNA reads were annotated, and DEGs were identified based on the following criteria: p-value <0.05 and |log_2_(fold-change)| ≥ 1.0. To assess gene expression patterns in coralthelial cells and HAECs, principal component analysis was performed using GraphPad Prism 9 (version 9.2.0; GraphPad Software, USA). DEGs enriched in the GO terms and pathways related to chemokines and cytokines in inflammation (p-value <0.05) were identified using the KEGG Orthology-Based Annotation System database (http://kobas.cbi.pku.edu.cn/kobas3).

### 4.12 Quantitative real-time PCR

RNA was extracted from cells using TRIzol reagent (Invitrogen, USA). cDNA was synthesized using PrimeScript™ RT Reagent Kit with gDNA Eraser (#RR047A; Takara, Japan) and an oligo (dT) primer. qRT-PCR assays were performed using 2 × Taq SYBR Green qPCR Premix (SQ101-01; Innovagene, China) on an Applied Biosystems QuantStudio™ 1 Real-Time PCR System (Thermo Fisher Scientific, USA). The total reaction volume was 10 μL, containing 5 μL of SYBR Premix Ex Taq (#RR390A; Takara, Japan), 0.4 μM each of forward and reverse primers, 1 μL of cDNA, and nuclease-free water to make up the remaining volume. The thermocycling program used for qRT-PCR was as follows: denaturation at 94 °C for 3 min; 40 cycles of denaturation at 94 °C for 10 s, annealing and extension at 60 °C for 40 s; and a melting cycle of 60 °C for 15 s. The primer sequences are listed below: IL-6, 5′-GCCTTCGGTCCAGTTGCCTTC-3′ (forward) and 5′-GTTCTGAAGAGGTGAGTGGCTGTC-3′ (reverse); CXCL8, 5′-CTCTCTTGG CAGCCTTCCTGATTTC-3′ (forward) and 5′-GGGGTGGAAAGGTTTGGAGTATGTC-3′ (reverse); MCP-1 (CCL2): 5′-ACCAGCAGCAAGTGTCCCAAAG-3′ (forward) and 5′-TTTGCTTGTCCAGGTGGTCCATG-3′ (reverse); SAR1B, 5′-GGGTGGACATGTTCA AGCTCGA-3′ (forward) and 5′-TCGCAACCTCTCTTCACTGATGG-3′ (reverse); RPL23, 5′-GGTGATGGCCACAGTCAAGA-3′ (forward), 5′-CGTTGTCGAATGACCACTGC-3′ (reverse); and GAPDH, 5′-TGTTCGTCATGGGTGTGAAC-3′ (forward) and 5′-ATGGCATGGACTGTGGTCAT-3′ (reverse). The mRNA levels of IL-6, CXCL8, and MCP-1 were normalized to GAPDH. The transcriptional level of each gene in coralthelial cells relative to HAECs was calculated using the 2^−ΔΔCT^ method.

### 4.13 Enzyme-linked immunosorbent assay

The concentrations of IL-6, MCP-1, and CXCL8 in the culture supernatants of HAECs and coralthelial cells on days 2, 5, 8, and 11 were measured using human IL-6, MCP-1, and CXCL8 ELISA Kits (#ELH-IL6, #ELH-MCP1, and #ELH-IL8; RayBio, USA), per the manufacturer's instructions.

### 4.14 Statistical analysis

Statistical significance between groups was determined by conducting Student's *t* tests or one-way analysis of variance using SPSS software (v26; IBM, USA). *P* < 0.05 was considered as the threshold for statistically significant differences.

## CRediT authorship contribution statement

**Ye Zeng:** Writing – review & editing, Writing – original draft, Visualization, Methodology, Investigation, Funding acquisition, Conceptualization. **Zhi Ouyang:** Visualization, Methodology, Investigation. **Yan Qiu:** Visualization, Methodology, Investigation. **Wenli Jiang:** Visualization, Methodology, Investigation. **Chen Jin:** Visualization. **Jian Zhong:** Visualization. **Linlu Jin:** Visualization. **Yixue Qin:** Visualization. **Yunran Zhao:** Visualization. **Xintong Zhou:** Visualization. **Xiaoheng Liu:** Supervision, Funding acquisition. **Bingmei M. Fu:** Writing – review & editing, Writing – original draft, Visualization, Supervision, Methodology, Funding acquisition, Conceptualization.

## Ethical approval

This study does not contain any studies with human or animal subjects performed by any of the authors.

## Declaration of competing interest

The authors declare the following financial interests/personal relationships which may be considered as potential competing interests: Ye Zeng reports financial support was provided by National Natural Science Foundation of China. Bingmei M. Fu reports financial support was provided by National Institutes of Health. Ye Zeng reports financial support was provided by the Natural Science Foundation of Sichuan Province. Ye Zeng has patent #ZL 2022 1 0412667.7 issued to China. If there are other authors, they declare that they have no known competing financial interests or personal relationships that could have appeared to influence the work reported in this paper.

## Data Availability

The data are available from the corresponding author on reasonable request.
