# Supplementary material for: Stacked human aortic endothelial cells induce atherosclerotic fatty streaks and release proinflammatory cytokines and chemokines
Source: Mechanobiol Med. 2026 Apr 27;4(2):100192. doi: 10.1016/j.mbm.2026.100192 (PMC13137019; doi:10.1016/j.mbm.2026.100192)
Supplement: Multimedia component 1 [file mmc1.docx]

Supplementary Materials for

Stacked Human Aortic Endothelial Cells Induce Atherosclerotic Fatty Streaks and Release Proinflammatory Cytokines

Ye Zeng*, Zhi Ouyang, Yan Qiu, Wenli Jiang, Chen Jin, Jian Zhong, Linlu Jin, Yixue Qin, Yunran Zhao, Xintong Zhou, Xiaoheng Liu, and Bingmei M. Fu*.

Correspondence to: ye@scu.edu.cn, and fu@ccny.cuny.edu

**This PDF file includes:**

Materials and Methods

Supplementary Text

Figures. S1 to S3

Tables S1

Materials and Methods

Detection of glycocalyx by wheat germ agglutinin (WGA) staining

Wheat germ agglutinin (WGA) binds to sialic acid and N-acetyl-D glucosamine of the glycocalyx. WGA staining has been used to detect and quantify the glycocalyx of cells. Cells were incubated with fluorescein isothiocyanate-labeled WGA (#L4895, FITC-WGA; Sigma Aldrich, USA) at 5 μg/mL for 1 h at room temperature in the dark. After washing in PBS three times for 5 min each, slides were counterstained with DAPI (Invitrogen, USA) for 5 min and observed under the confocal laser scanning microscopy. The orthogonal view of the WGA stack was performed using Zen blue 3.6 software (Zeiss, Germany). The quantification analyses were performed using ImageJ software.

Toluidine Blue staining

The sulfate and/or carboxylic groups in mucopolysaccharides (acidic glycosaminoglycans) and proteoglycan complexes were stained by Toluidine Blue staining (#G3660, Solarbio, China). Cells were washed three times with PBS, fixed with 95% ethanol for 15 s, rinsed with deionized water, stained with 500 μL Toluidine Blue solution for 5 min and maintained in deionized water for 15 min. After rinsing with deionized water, the samples were observed under an inverted phase contrast microscope.

Periodic Acid-Schiff (PAS) staining

A PAS staining kit (#G1360, Solarbio, China) was used to detect the carbohydrate aggregation. Cells were washed three times with PBS, fixed with the PAS fixative (Reagent A) for 15 min, washed three times with deionized water and air-dry, and treated with the oxidant (Reagent B) at room temperature for 15 min. Then, cells were rinsed with deionized water, stained in the Schiff reagent (Reagent C) at room temperature for 20 min in dark, rinsed with the sodium sulfite solution (Reagent D) twice for 2 min each, and then redyed with the Mayer Hematoxylin staining solution for 2 min, rinsed with deionized water and observed.

Picrosirius Red Staining

Picrosirius Red staining (#S8060, Solarbio, China) was performed for collagen detection. Cells were washed three times with PBS, fixed in 4% paraformaldehyde for 10 min, washed three times with deionized water, stained with 0.1% Sirius Red solution for 30 min, rinsed with deionized water and observed.

Alizarin red S staining

Alizarin red S staining (#G1452, Solarbio, China) was performed to detect the calcium deposition. Cells were washed three times with PBS, fixed in 4% paraformaldehyde for 10 min, washed three times with PBS, stained with 0.2% Alizarin Red S solution for 20 min, rinsed with PBS and observed.

Assessment of spatial confinement and apoptosis (Hoechst 33342 and 7-AAD staining)

To evaluate the spatial distribution and viability of the cultured cells, Hoechst 33342 (#T5840, TargetMol, China) and 7-Aminoactinomycin D (7-AAD; #P-CA-162, Procell, China) staining were performed. Briefly, the culture medium was removed, and the cells were washed three times with PBS. The cells were then incubated with Hoechst 33342 (to visualize all nuclei) and 7-AAD (to detect apoptotic or dead cells with compromised membrane integrity) for 10-15 minutes at room temperature in the dark. Following incubation, the cells were washed with PBS and immediately observed under an inverted fluorescence microscope. Corresponding bright-field images were simultaneously captured to evaluate the overall cellular morphology and spatial confinement.

Senescence-associated β-galactosidase (SA-β-gal) staining

Cellular senescence was evaluated using a SA-β-gal staining kit (#C0602, Beyotime, China). Cells were washed three times with PBS and fixed with the provided fixative solution for 15 minutes at room temperature. After washing three times with PBS to remove the residual fixative, the cells were incubated with the SA-β-gal working solution overnight at 37°C in a dry incubator (without CO_2_) protected from the light. Following the incubation, the staining solution was discarded, and the cells were washed with PBS before being observed and imaged under an inverted phase contrast microscope.

Supplementary Text

In addition to lipid and foam cells, collagens and ECM contribute to the intimal thickening ^1^. Collagens comprise a major portion of proteins in the ECM found in the atherosclerotic plaque. In the lesion-free intima from 23 human subjects, out of 100 mg dry matter, 2.5 mg is mucopolysaccharide, 25 mg is collagen, and 9.8 mg is lipid. In contrast, in the fatty streak region, there is an increase of 0.33 mg in mucopolysaccharide, paralleled by an increase of 3.3 mg in collagen but 22 mg in lipid per 100 mg dry tissue ^2^. VSMCs are thought to be the main cell type responsible for collagen synthesis in the vessel wall. VSMCs orchestrate the assembly of type I collagen fibril that is linked to the cytoskeleton ^3^. Meanwhile, type I collagens are usually present in the diseased vessels and support the proliferation of VSMCs ^4^.

Reduced glycosaminoglycans (GAGs) and enhanced proteoglycans in coralthelial cells.

The endothelium is intact over large lesions. It is believed that the ECs overlying the lesion are abnormally large while they lose argyrophilic properties with unclear cell boundaries ^5^. Loss of silver precipitation at the lesion areas is thought to be associated with the degradation of glycocalyx during atherosclerotic lesion development ^5,6^. Glycocalyx plays a critical role in vascular homeostasis ^7^, and its coverage and thickness are reduced even before the atherosclerotic plaque formation ^8^. At plaque sites, particularly at bifurcations, glycocalyx is markedly diminished on ECs ^9^. The degradation of the glycocalyx is closely associated with increased artery stiffness caused by hypertension and aging ^10,11^. It should be noted, however, that an early report demonstrated that the concanavalin A reactive coat (glycocalyx) over endothelium varies in thickness during lesion development. Initially, it becomes thicker (0.2-0.6 µm), but eventually becomes amorphous depositions adhering to the denuded endothelial surface ^12^.

The glycocalyx at the cells was examined by staining with wheat germ agglutinin (WGA) and toluidine blue (**Figs. S3A-D**). WGA specifically binds to N-acetyl-D-glucosamine and N-acetyl-D-neuraminic acid of GAGs ^13^. Toluidine blue is a cationic dye with high affinity with the sulfate and/or carboxylic groups of mucopolysaccharides (acid GAGs) and proteoglycan complexes ^14^. While WGA-stained GAGs are distributed on the surface of HAECs in the monolayer (left two panels in **Fig. S3A**), GAGs are largely reduced in the coralthelial cells and dispersed in their cytoplasm (right two panels in **Fig. S3A**). The MFI of GAGs at the coralthelial cells is only 32.77 ± 8.85% of HAECs in the monolayer (**Fig. S3B**). However, the extracellular matrix proteoglycan complexes are significantly increased in the coralthelial cells (**Fig. S3C**), compared with that in the HAECs of the monolayer, 17.95 ± 1.48% vs. 7.06 ± 1.56% (**Fig. S3D**). The residual proteoglycans are predominantly distributed in the coral-like structures.

Enriched glycogen, collagens, and calcium in coralthelial cells.

The accumulated periodic acid Schiff (PAS) positive materials in the coralthelial cells are significantly increased compared to those in the HAECs of the monolayer, 32.53 ± 2.96% vs. 7.79 ± 0.76% (**Figs. S3E, F**), along with much more toluidine blue positive materials (**Figs. S3C and D**), supporting that the glycogen and residual proteoglycans deposits are aggregated in the coral-like structures. Picrosirius red staining was performed to quantify collagen deposition (**Fig. S3G**). Coralthelial cells appear to have more intense staining of collagen, 27.47 ± 2.75% vs. 4.62 ± 2.41%, compared to HAECs in the monolayer (**Fig. S3H**). Collagen fibrils appear to scaffold the coral-like structures. Calcium and mineralization within cells were assessed by Alizarin red staining (**Fig.S3I**). Substantially more Alizarin red staining was seen in coralthelial cells than that in HAECs of the monolayer, 23.35 ± 4.13% vs. 2.37 ± 0.55% (**Fig. S3J**). Like proteoglycans, glycogen and collagen, calcium is also distributed in the coral-like structures of coralthelial cells. It appears that the coralthelial cells (or transformed HAECs) can synthesize collagen fibrils as a supporting network for the accumulation of lipid droplets, proteoglycans, glycogens and calcium to form the fatty streak-like structure.

Our findings suggest that HAEC-derived coralthelial cells also produce much more collagens than original HAECs. The collagens form an ECM scaffold, facilitating the assembly of coralthelial cells into fatty streak-like structure. Proteoglycans, glycogen, and calcium were also abundant in these structures. Proteoglycans ^15^, collagen type I ^16^ and sulfated glycosaminoglycans ^17^, which enhance intimal retention of plasma LDLs, probably enter the autophagic vacuoles to mix with droplets. We also observed autophagic vacuoles filled with moderately electrodense flocculent matrixes and lipid droplets in the coralthelial cells.

**Figure S1. Spatial confinement of stacked HAECs and assessment of cellular status after 11-day continuous culture.**

(Top) Optical and Hoechst 33342 fluorescent images show that the stacked HAECs (Pile) are confined to an elongated ridge-like region, confirming that they do not proliferate to cover the entire P35 culture dish or reach an over-confluence.

(Bottom) Representative images of senescence-associated β-galactosidase (SA-β-gal) and 7-AAD staining reveal no obvious cellular senescence or apoptosis in the cultured cells forming the monolayer. Together, these results verify that the 11-day continuous culture protocol without medium replacement maintains cell health and does not induce cytotoxicity related to nutrient depletion in either the stacked or monolayer conditions.


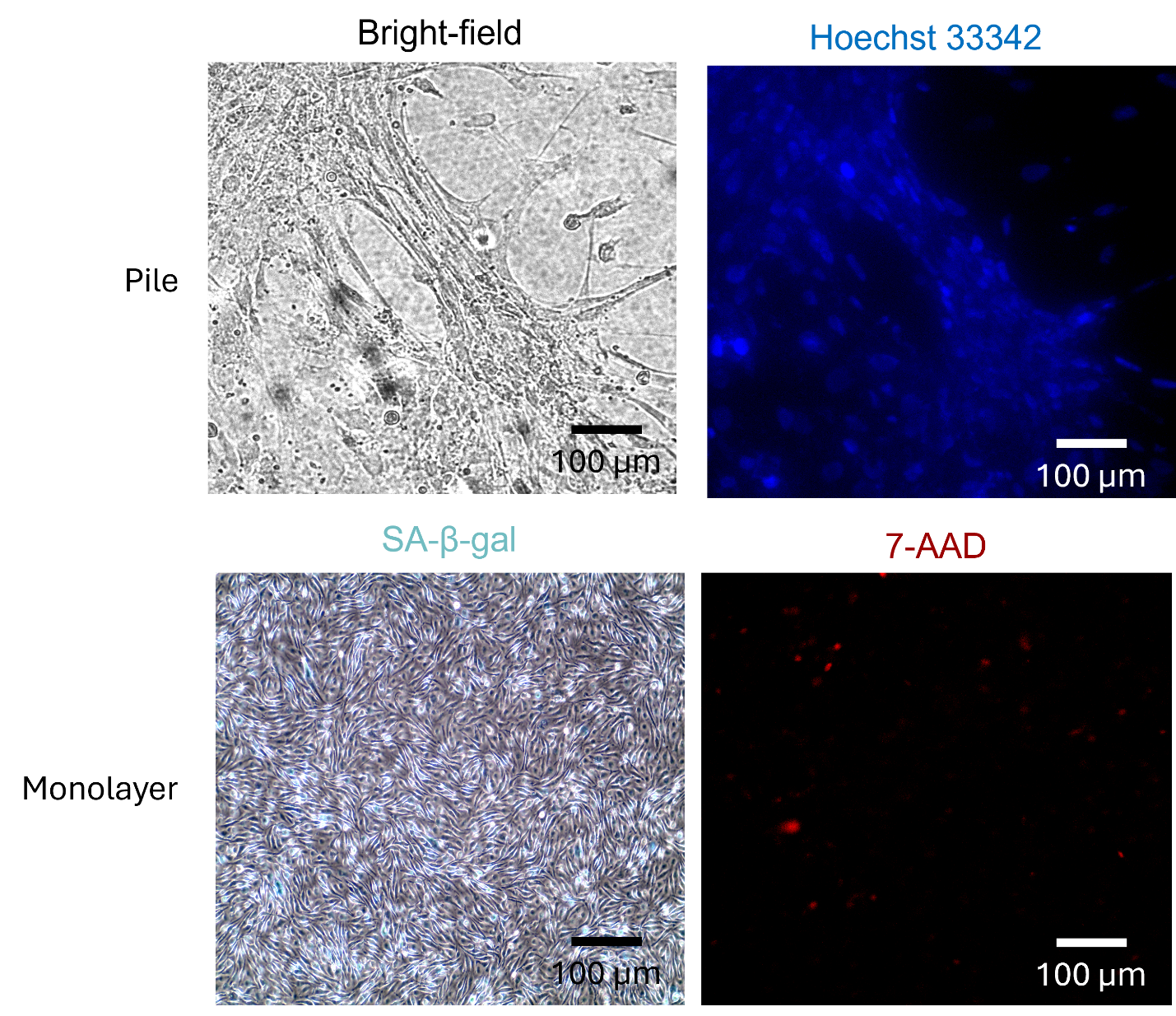


Figure S2.

**The interference efficiency of the small interfering RNAs for SAR1B and RPL23. SAR1B-3 and RPL23-3 were used in following experiments.** Mean ± standard deviation (SD); * P<0.05, ***P<0.001 vs. siNC.

Figure S3.

**Distribution of glycocalyx, glycogen, collagens and calcium in HAECs in the monolayer and coralthelial cells in the pile.** After culturing for 11 days, HAECs from the monolayer and coralthelial cells from the pile were stained with WGA for surface glycosaminoglycans (**A**), toluidine blue for mucopolysaccharides and proteoglycan complexes (**C**), Periodic Acid-Schiff (PAS) for carbohydrate aggregation (**E**), Picrosirius red for collagen (**G**), and Alizarin red for calcium and mineralization (**I**). The dashed circle represents the stacked cells in Fig.S2A. The corresponding mean fluorescence intensity (MFI) of these stains is shown in (**B, D, F, H, J**), respectively. Mean ± SD; **P<0.01, ***P<0.001.

**
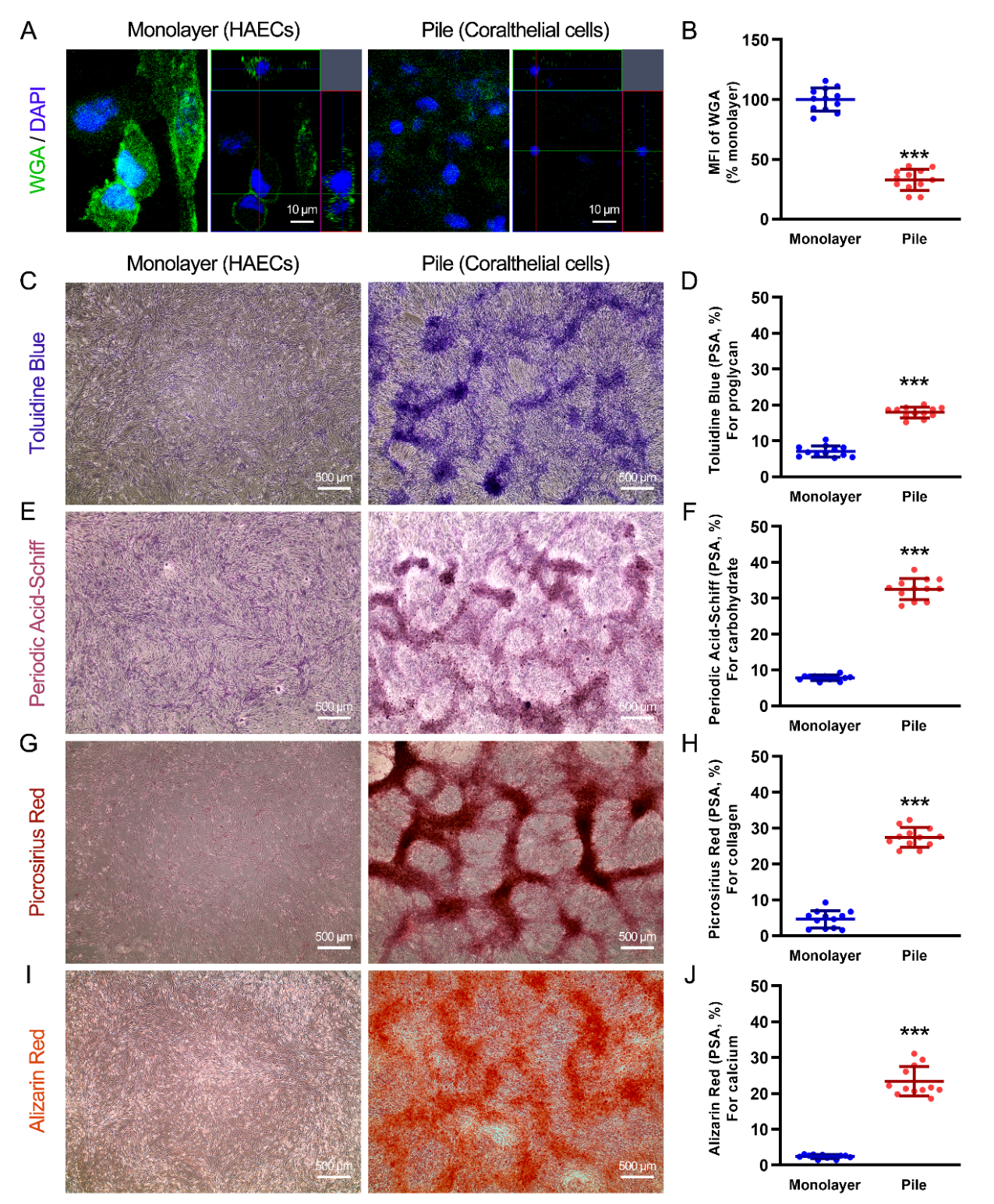
**

Table S1.

siRNA sequences for RNA interference.

| Primer |  | Sequence (5’ - 3’) |
| --- | --- | --- |
| ^1^ si*SAR1B* | #1 | F: GCAUGACGUUUACAACUUU  R: AAAGUUGUAAACGUCAUGC |
|  | #2 | F: GGAGUAUAUCUCUGAAAGA  R: UCUUUCAGAGAUAUACUCC |
|  | #3^&^ | F: GGAUUGUGCAGACCACGAA  R: UUCGUGGUCUGCACAAUCC |
| ^2^ si*RPL23* | #1 | F: CAACGAAAGUCAUACCGUA(dT)(dT)  R: UACGGUAUGACUUUCGUUG(dT)(dT) |
|  | #2 | F: GAGUCAUAGUGAACAAUAA(dT)(dT)  R: UUAUUGUUCACUAUGACUC(dT)(dT) |
| ^3^ siNC | #3^&^ | F: CGGUAGGAGCUGUAAUCAA(dT)(dT)  R: UUGAUUACAGCUCCUACCG(dT)(dT) |
|  |  | F: UUCUCCGAACGUGUCACGU(dT)(dT)  R: ACGUGACACGUUCGGAGAA(dT)(dT) |

^1^ si*SAR1B*, small interfering RNA for *SAR1B*. ^2^ si*RPL23*, small interfering RNA for *RPL23.* ^3^ siNC, small interfering RNA for negative control. ^&^Used in the following experiments.

**References**

1 Malcom, G. T. *et al.* Associations of arterial tissue lipids with coronary heart disease risk factors in young people. *Atherosclerosis* **203**, 515-521 (2009). <https://doi.org/10.1016/j.atherosclerosis.2008.07.002>

2 Smith, E. B. The influence of age and atherosclerosis on the chemistry of aortic intima. 2. Collagen and mucopolysaccharides. *J Atheroscler Res* **5**, 241-248 (1965). <https://doi.org/10.1016/s0368-1319(65)80065-5>

3 Li, S., Van Den Diepstraten, C., D'Souza, S. J., Chan, B. M. & Pickering, J. G. Vascular smooth muscle cells orchestrate the assembly of type I collagen via alpha2beta1 integrin, RhoA, and fibronectin polymerization. *The American journal of pathology* **163**, 1045-1056 (2003). <https://doi.org/10.1016/s0002-9440(10)63464-5>

4 Koyama, H., Raines, E. W., Bornfeldt, K. E., Roberts, J. M. & Ross, R. Fibrillar collagen inhibits arterial smooth muscle proliferation through regulation of Cdk2 inhibitors. *Cell* **87**, 1069-1078 (1996). <https://doi.org/10.1016/s0092-8674(00)81801-2>

5 Reidy, M. A. & Bowyer, D. E. Scanning electron microscope studies of rabbit aortic endothelium in areas of haemodynamic stress during induction of fatty streaks. *Virchows Archiv. A, Pathological anatomy and histology* **377**, 237-248 (1978). <https://doi.org/10.1007/bf00426933>

6 Goode, T. B., Davies, P. F., Reidy, M. A. & Bowyer, D. E. Aortic endothelial cell morphology observed in situ by scanning electron microscopy during atherogenesis in the rabbit. *Atherosclerosis* **27**, 235-251 (1977). <https://doi.org/10.1016/0021-9150(77)90061-2>

7 Weinbaum, S., Cancel, L. M., Fu, B. M. & Tarbell, J. M. The Glycocalyx and Its Role in Vascular Physiology and Vascular Related Diseases. *Cardiovascular engineering and technology* **12**, 37-71 (2021). <https://doi.org/10.1007/s13239-020-00485-9>

8 Bar, A. *et al.* Degradation of Glycocalyx and Multiple Manifestations of Endothelial Dysfunction Coincide in the Early Phase of Endothelial Dysfunction Before Atherosclerotic Plaque Development in Apolipoprotein E/Low-Density Lipoprotein Receptor-Deficient Mice. *J Am Heart Assoc* **8**, e011171 (2019). <https://doi.org/10.1161/jaha.118.011171>

9 Cancel, L. M., Ebong, E. E., Mensah, S., Hirschberg, C. & Tarbell, J. M. Endothelial glycocalyx, apoptosis and inflammation in an atherosclerotic mouse model. *Atherosclerosis* **252**, 136-146 (2016). <https://doi.org/10.1016/j.atherosclerosis.2016.07.930>

10 Mahmoud, M., Cancel, L. & Tarbell, J. M. Matrix Stiffness Affects Glycocalyx Expression in Cultured Endothelial Cells. *Frontiers in cell and developmental biology* **9**, 731666 (2021). <https://doi.org/10.3389/fcell.2021.731666>

11 Mahmoud, M. *et al.* The glycocalyx core protein Glypican 1 protects vessel wall endothelial cells from stiffness-mediated dysfunction and disease. *Cardiovasc Res* **117**, 1592-1605 (2021). <https://doi.org/10.1093/cvr/cvaa201>

12 Weber, G., Fabbrini, P. & Resi, L. On the presence of a concanavalin-A reactive coat over the endothelial aortic surface and its modifications during early experimental cholesterol atherogenesis in rabbits. *Virchows Archiv. A, Pathology. Pathologische Anatomie* **359**, 299-307 (1973). <https://doi.org/10.1007/bf00548601>

13 Wright, C. S. Structural comparison of the two distinct sugar binding sites in wheat germ agglutinin isolectin II. *J Mol Biol* **178**, 91-104 (1984). <https://doi.org/10.1016/0022-2836(84)90232-8>

14 Vidal, B. C. & Mello, M. L. S. Toluidine blue staining for cell and tissue biology applications. *Acta Histochem* **121**, 101-112 (2019). <https://doi.org/10.1016/j.acthis.2018.11.005>

15 Vijayagopal, P. *et al.* Lipoprotein-proteoglycan complexes induce continued cholesteryl ester accumulation in foam cells from rabbit atherosclerotic lesions. *The Journal of clinical investigation* **91**, 1011-1018 (1993). <https://doi.org/10.1172/jci116257>

16 Ho-Tin-Noé, B. *et al.* Cholesterol crystallization in human atherosclerosis is triggered in smooth muscle cells during the transition from fatty streak to fibroatheroma. *The Journal of pathology* **241**, 671-682 (2017). <https://doi.org/10.1002/path.4873>

17 Tovar, A. M., Cesar, D. C., Leta, G. C. & Mourão, P. A. Age-related changes in populations of aortic glycosaminoglycans: species with low affinity for plasma low-density lipoproteins, and not species with high affinity, are preferentially affected. *Arteriosclerosis, thrombosis, and vascular biology* **18**, 604-614 (1998). <https://doi.org/10.1161/01.atv.18.4.604>
